# Effectiveness of Interactive Tools in Online Health Care Communities: Social Exchange Theory Perspective

**DOI:** 10.2196/21892

**Published:** 2021-03-12

**Authors:** Dixuan Ren, Baolong Ma

**Affiliations:** 1 School of Management and Economics Beijing Institute of Technology Beijing China

**Keywords:** efforts, income, privacy disease, platform, social exchange

## Abstract

**Background:**

Although the COVID-19 pandemic will have a negative effect on China’s economy in the short term, it also represents a major opportunity for internet-based medical treatment in the medium and long term. Compared with normal times, internet-based medical platforms including the Haodf website were visited by 1.11 billion people, the number of new registered users of all platforms increased by 10, and the number of new users’ daily consultations increased by 9 during the pandemic. The continuous participation of physicians is a major factor in the success of the platform, and economic return is an important reason for physicians to provide internet-based services. However, no study has provided the effectiveness of interactive tools in online health care communities to influence physicians’ returns.

**Objective:**

The effect of internet-based effort on the benefits and effectiveness of interactive effort tools in internet-based health care areas remains unclear. Thus, the goals of this study are to examine the effect of doctors’ internet-based service quality on their economic returns during COVID-19 social restrictions, to examine the effect of mutual help groups on doctors’ economic returns during COVID-19 social restrictions, and to explore the moderating effect of disease privacy on doctors’ efforts and economic returns during COVID-19 social restrictions.

**Methods:**

On the basis of the social exchange theory, this study establishes an internet-based effort exchange model for doctors. We used a crawler to download information automatically from Haodf website. From March 5 to 7, 2020, which occurred during the COVID-19 pandemic in China, cross-sectional information of 2530 doctors were collected.

**Results:**

Hierarchical linear regression showed that disease privacy (β=.481; *P*<.001), reputation (β=.584; *P*<.001), and service quality (β=.560; *P*<.001) had a significant positive effect on the economic returns of the physicians. The influence of mutual help groups on earnings increases with an increase in the degree of disease privacy (β=.189; *P*<.001), indicating that mutual help groups have a stronger effect on earnings when patients ask questions about diseases regarding which they desire privacy.

**Conclusions:**

For platform operators, the results of this study can help the platform understand how to improve doctors’ economic returns, especially regarding helping a specific doctor group improve its income to retain good doctors. For physicians on the platform, this study will help doctors spend their limited energy and time on tools that can improve internet-based consultation incomes. Patients who receive internet-based health care services extract information about a doctor based on the doctor’s internet-based efforts to understand the doctor’s level of professionalism and personality to choose the doctor they like the most. The data used in this study may be biased or not representative of all medical platforms, as they were collected from a single website.

## Introduction

### Background

Since December 2019, the highly contagious novel coronavirus pneumonia (COVID-19) has been spreading within and beyond China [1]. The first case in China was identified in Wuhan City, Hubei Province, in early December [2]. To contain the outbreak, Wuhan imposed a quarantine from 10 AM on January 23, 2020, onward [3]. Most cities in Hubei Province were also quarantined, restricting people from traveling out of their own cities. People in proximity can easily transmit COVID-19; thus, social (ie, physical) distancing is an important measure to reduce its spread [4]. By February 2020, China had introduced travel bans and quarantine measures, closed public services, and canceled social events to contain COVID-19. COVID-19 also endangers people undergoing in-person care at hospitals as patients with different diseases gather in hospitals; therefore, the government stopped offline care to avoid spreading COVID-19 between doctors and patients [5]. However, medical consultations with patients who do not have COVID-19 remained a challenge during the pandemic.

The obvious answer to this challenge is online health care community (OHC), in which doctors can provide primary care for patients via internet-based platforms. This method can effectively prevent COVID-19 infections, which are deadly and highly transmissible [6,7]. Moreover, patients who do not have emergent diseases can receive doctor consultations. China Medical and Health Service System Planning (2015-2020) promulgated by the State Council notes that it is necessary to actively use the internet, cloud computing, and other information technologies to transform the existing health service model to benefit Chinese health service needs. Undoubtedly, the OHC will usher in unprecedented development opportunities as the government actively guides and supports the management of COVID-19 pandemic [8]. Compared with traditional offline health care, the OHC can prevent COVID-19 infections and operate outside of typical health care restrictions regarding time and space, as patients can communicate with doctors and obtain health information anytime and anywhere [6]. Therefore, the use of internet-based platforms to offer primary patient care provides a new and beneficial method to effectively allocate medical resources, improve doctor-patient relationships, and reduce medical costs and patient waiting time.

The prosperity of the OHC cannot be achieved without the continued participation and efforts of doctors, particularly high-status doctors who have always been a scarce medical resource. In the short term, doctors may want to gain reputation; however, in the long term, they still want to earn money [9]. Performance output is closely related to a person’s effort at work [10]. The OHC provides convenience for patients and should consider the economic returns of doctors to encourage doctors to actively participate, change their methods, and reasonably allocate their own energy and time both online and offline. An effort has been defined as the accumulated amount of time invested, energy spent, or activity by which work is accomplished [10-12] and is composed of three factors: direction (working smart), level (working hard), and persistence (in terms of the amount of time spent working and continuing to try to achieve a goal in the face of failure). Doctors’ efforts aim for patients to perceive the professional ability and attitude of service doctors, then patients feel satisfaction and hope for gaining further internet-based services, and the doctors’ webpages are visited frequently, ultimately increasing the economic return of the doctors. The OHC mainly relies on the exchange of health knowledge and information between doctors and patients [13]. Therefore, the relationship between doctors’ internet-based efforts and their economic returns is of great significance for OHC development. Whether health care workers can modify their traditional health care methods and actively participate in and adapt to the new health care model of the OHC remains to be determined. This study primarily reviews existing studies from the perspective of social exchange theory and the relationship between effort and earnings.

### Literature Review

#### Social Exchanges Between Patients and Doctors

Social exchange theory describes the relationship among people from the perspective of benefits and costs. Individual behavior typically tries to maximize benefits by paying as little as possible [14], finding the optimal solution between costs and benefits. The participation of doctors in the OHC can be considered as a social exchange behavior that is described by social exchange theory [15] rather than a purely economic exchange. Social exchange theory advocates the use of economic methods to analyze noneconomic social behaviors and has been widely used to understand the dynamic exchange processes in social relationships [16]. On the basis of a systematic review of the literature on social exchange theory, 2 critical features of social exchange have been clarified: (1) dynamic interaction behaviors (ie, repeated exchange actions) and (2) the power in structural relationships (ie, the power to maintain the interdependent relationship among the exchangers in a social exchange) [17].

#### Relationship Between Efforts and Earnings

For service areas, efforts can enhance consumers’ perceptions of the quality of products. Employees’ work efforts influence customer satisfaction, and customers perceive that the harder employees work, the more satisfied they will be of the services they receive [18]. Efforts in the health consultation market are perceived as an exchange of resources in which doctors actively demonstrate their ability to attract more attention and obtain more consultation [18,19]: the harder employees work, the better their performance will be. There is a positive correlation between employee effort and job performance, and work effort level is one of the determinants of job performance [20,21]. Individuals or organizations with a strong sense of reciprocity are more willing to share knowledge and believe that knowledge sharing will yield certain personal rewards [22,23]. In the OHC market, there is a strong link between doctors’ internet-based efforts and their internet-based rewards.

### Research Model

The professional capital used by doctors to participate in knowledge exchange describes the attitudes of interaction between the doctors and patients during social exchanges as well as the doctors’ professional commitment (eg, enthusiasm, responsibility, and morality) [24]. The professional capital of doctors can be directly observed by patients through doctors’ effort behavior, which is shown in a series of dynamic interactive therapeutic behaviors because the interaction behavior of doctors is guaranteed by their ability to act independently and a set of rules of commitment [25]. As previously mentioned, studies have shown that effort has a positive effect on performance. The OHC is a typical information asymmetry market, and doctors know their professional abilities better than patients [9]. As recipients, patients interpret doctors’ efforts and adjust their purchasing behavior accordingly. Patients who receive a doctor’s effort are typically more likely to buy health care products than patients who do not receive it. On the basis of social exchange theory, doctors should provide information about their medical ability to users through effort behaviors to improve their earnings: the more doctors work, the more benefits they can obtain. The internet-based efforts of doctors can influence patient selection based on the social exchange function and affect the internet-based economic return and offline referrals of doctors. Patients believe that doctors who make more efforts possess higher service quality and therefore have a higher probability of being selected; thus, doctors gain higher economic returns when they put forth more effort.

Person efforts have not been considered in previous studies on the OHC market. There is a strong link between effort and performance, in which the OHC platform only supplies 2 interactive tools of effort between physicians and patients. However, effectiveness of interactive effort tools in internet-based health care areas remain unclear. Thus, we aim to (1) examine the effect of doctors’ internet-based service quality on their economic returns during COVID-19 social restrictions, (2) examine the effect of mutual help groups built on their economic returns during COVID-19 social restrictions, and (3) explore the moderating effect of disease privacy on individual efforts and economic returns during COVID-19 social restrictions.

**Figure 1:**
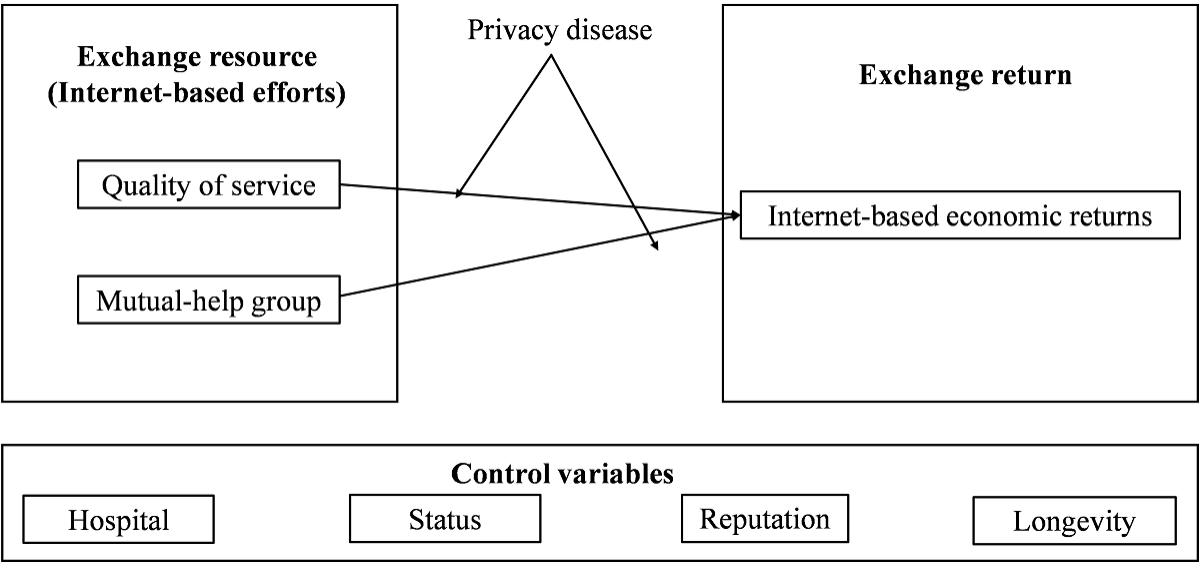
Exchange model describing doctors’ efforts and returns.

On the basis of social exchange theory, doctors’ participation in the OHC is a process in which professional capital participates in social exchange for economic returns. The research model is illustrated in Figure 1.

## Methods

### Study Design

In the OHC, patients can assess the value of doctors through the distribution and frequency of interactions with doctors (eg, the doctors’ replies and the mutual help groups) to describe doctors’ ability and willingness to work [26]. In this study, the benefit of internet-based consultation refers to the financial return that doctors obtain after performing certain tasks that expend time and energy. In combination with social exchange theory and the scenario investigated in this study, this study identifies two effort tools of doctor-patient interaction: quality of service and mutual help groups.

Interaction between sellers and consumers increases sellers’ trust and promotes consumers’ behavioral intentions [27]. Positive interactions that allow patients to feel the doctor’s efforts and service attitudes online can help overcome distrust between doctors and patients.

OHCs are a collection of virtual discussion groups in which members can share feelings, knowledge, and experience about the health topics of their interests [28]. Doctors provide communication opportunities for patients with similar diseases. Patients can find valuable resources through a doctor’s effort in a series of interactions in OHCs. Exchange behaviors are used to bridge doctors’ efforts and patients’ rewards.

Different disease types have different psychological characteristics, cognitive needs, and patient involvement [29]. Privacy diseases refer to diseases that patients are reluctant to disclose to others, as disclosure of relevant information about these diseases will lead to a series of consequences, such as patients' mental stress, love discrimination, disease discrimination, work pressure, and social stigma [16]. Thus, OHCs provide a channel for privacy patients to acquire medical knowledge. Internet-based doctors’ efforts (eg, the quality of service and mutual help groups) can effectively eliminate patients’ concerns; thus, the positive effect on economic returns is stronger.

Thus, we hypothesize the following:

- Hypothesis 1 (H1): The service quality of physicians in OHCs is positively associated with the economic returns of doctors.
- Hypothesis 2 (H2): Mutual help groups in OHCs are positively associated with the internet-based economic returns of doctors.
- Hypothesis 3 (H3): The degree of disease privacy positively regulates the effect of physicians’ service quality on revenue from internet-based consultations.
- Hypothesis 4 (H4): The degree of disease privacy positively regulates the influence of mutual help groups on the revenue of internet-based consultations.

### Data Collection

This study collected data from haodf website for analysis. Haodf website is a typical internet-based health consulting service platform in China that has a long history of establishment, mature operation, high user coverage, high doctor activity, and high-quality hospital. Therefore, Haodf website was investigated in this study. By March 20, 2020, 591,832 doctors from 9614 hospitals had registered on the haodf website. Thus, haodf is a representative internet-based health advisory platform.

We used a crawler to download information automatically from Haodf website. From March 5, 2020, data collection lasted for 2 days and involved 2530 doctors. We collected the service quality, groups, status, and other relevant information for each doctor in the data set.

### Measures

Physician engagement in OHCs is typically a form of social exchange with patients, which is characterized by structural power and dynamic interactions [15]. Income is important for social exchange. The product of the number of doctors’ services and the unit price of consultation was used to determine the internet-based economic rewards.

Social exchange is a two-way transaction: “there must be both pay and return” [30]. Doctors can use two tools to show their efforts on the Haodf website: the doctors’ quality of service and the mutual help group. The Haodf website provides patients with the ability to consult doctors [31]. This study uses the number of doctors who respond to user inquiries on the platforms as a variable of doctors’ service quality. We assessed mutual help groups using the number of groups in which patients who consulted a doctor was assigned to different topic groups based on their disease characteristics. Communication in the same topic group occurred between the patients and doctors or patients with similar diseases. Doctors could provide service information and advice to the group. Disease privacy refers to a patient’s reluctance to tell others or disclose that they have a disease [9]. Hepatitis B is highly contagious, and the disclosure of disease-related information may cause discrimination. Therefore, hepatitis B was investigated in this study as a disease with a high degree of privacy.

On the basis of the research model and hypotheses, the economic returns of internet-based care are affected by the internet-based efforts of doctors (quality of service and mutual help groups) and the degree of disease privacy. In addition, economic returns may be affected by other factors. First, the professional title of doctors affects doctors’ internet-based economic returns. High-status doctors have higher priorities and privileges. For example, patients are always more willing to see the chief physician regardless of the doctors’ professional level. Second, doctors’ profits are affected by their reputation. Reputation in the internet-based market is primarily described through evaluation and feedback. Sellers with a better reputation have higher price premiums [32]. Finally, customers are more willing to visit century-old stores because they assume that stores with a longer operation time can deliver good service quality to customers. Similarly, if the doctor’s home page has been operational longer and updated recently, the doctor’s service quality is more likely to be recognized by patients. Therefore, city level, hospital level, doctor title, reputation, and internet-based service life was considered as control variables in this study.

Variables measured in this study are shown in Table 1.

**Table 1:** Measurement of variables.

Variables		Measurement
**Dependent variables**		
	Internet-based economic rewards	The product of the number of doctors’ services and the unit price of consultation.
**Independent variables**		
	Doctors’ quality of service	Sum up the number of doctors’ answers from the patient consultation area of the doctor’s home page.
	Mutual help groups	Grab the count directly from the topic page of the doctor’s personal home page.
**Moderator variables**		
	Privacy disease	Hepatitis B
**Control variables**		
	Hospital level	On the basis of the classification of 3 levels, 1 is the tertiary hospital, 2 is a secondary hospital, and 3 is the class-1 hospital. The higher the number, the higher the hospital’s level.
	Status	There are 5 grades: the chief physician is fifth grade, the associate chief physician is fourth grade, the attending physician is third grade, the resident physician is second grade, and other doctors are first grade. The higher the number, the higher the doctor’s rank.
	Reputation	As the value range of these 3 indicators, including votes, letter of thanks, and gifts, vary, they are averaged after standardization to measure the reputation of doctors.
	Longevity	The number of years between the time the doctor’s home page was created and the time the data were collected.

### Statistical Results

Quantitative survey data were analyzed using SPSS (version 25.0; IBM Corporation). Table 2 shows the descriptive statistical results for all the variables. Associate chief physicians and chief physicians account for approximately half of all online doctors [33]. As shown in Table 2, the mean of doctor status was 4.52, indicating that the status of online doctors was above that of associated chief physicians. The average hospital rating was 2.99, indicating that the hospital where the online doctor worked offline was above level 2. Reputation is the average of the 3 indicators standardized (thank you letters, votes, and gifts); thus, its mean is 0. The average age of the doctor’s home page was 8.46 years, the average number of online responses per doctor was 10,436.90, and the average number of mutual help groups built per doctor was 63.13.

**Table 2:** Results for descriptive statistics.

Variables	Value, mean (SD)	Minimum value	Maximum value
Returns	14,974.64 (32,179.683)	0	270,660
Quality	10,436.90 (10,427.042)	946	78,331
Groups	63.13 (152.230)	0	1651
Privacy	0.20 (0.399)	0	1
Hospital	2.99 (0.140)	1	3
Status	4.52 (0.721)	1	5
Reputation	0.15 (0.965)	−0.78	4.83
Reputation 1	120.20 (126.967)	0	75
Reputation 2	260.82 (254.238)	7	1421
Reputation 3	380.23 (466.302)	11	2785
Longevity	8.46 (2.419)	1.76	11.04

## Results

To ensure that multicollinearity does not confound the regression results, this study first examined the variance inflation factors (VIFs) among variables. As all VIFs are lower than 5, no serious multicollinearity exists among the independent variables [34,35].

In this study, hierarchical regression analysis was used to examine the effect of the quality of service and mutual help groups on online economic rewards as well as the moderating effect of disease privacy. Considering the research design, the hierarchical regression analysis method is more suitable for this study because this study conducts a separate analysis of the variables of each layer to determine the differences among them. The purpose of the hierarchical regression analysis method is to investigate the contribution of the variable to the regression equation when the contributions of other variables are excluded. The regression results of the hierarchical regression analysis in this study are presented in Table 3.

**Table 3:** Regression analysis.

Variables		Dependent variable: internet-based economic returns, nonstandardized coefficients (SE)		
		Model 1	Model 2	Model 3
**Control variables**				
	Privacy	0.481^a^ (0.127)	0.332^b^ (0.114)	0.313^b^ (0.108)
	Hospital	−0.072 (0.362)	−0.008 (0.321)	−0.003 (0.299)
	Status	0.087 (0.079)	0.124 (0.070)	0.120 (0.066)
	Reputation	0.584^a^ (0.054)	0.206^a^ (0.067)	0.305^a^ (0.065)
	Longevity	−0.019 (0.024)	−0.024 (0.021)	−0.023 (0.020)
**Independent variables**				
	Quality	N/A^c^	0.560^a^ (0.069)	0.408^a^ (0.069)
	Groups	N/A	−0.062 (0.056)	−0.082 (0.052)
	Quality × privacy	N/A	N/A	0.066 (0.047)
	Groups × privacy	N/A	N/A	0.189^a^ (0.053)

^a^*P*<.001.

^b^*P*<.01.

^c^N/A: not applicable.

Model 1 only contains control variables, and the results show that the degree of disease privacy (*P*<.001) and reputation (*P*<.001) have a significant positive effect; thus, doctors who provide medical services for patients with a high degree of disease privacy can obtain more economic returns. Channels between online and offline have substitutes and synergies [36,37]. Patients with nonprivacy diseases can go to hospitals without any concern, providing an alternative to internet-based medical services. However, patients with privacy diseases do not wish to go to hospitals for treatment and tend to use internet-based medical services. Therefore, OHCs tend to have patients with privacy diseases [9]. The internet-based reputation of doctors has a positive effect on doctors’ internet-based incomes. A good reputation indicates that patients recognize the quality of service and indicates a lower risk of service quality. At a significance level of *P*<.05, the hospital level (*P*=.84), doctors’ status (*P*=.27), and longevity (*P*=.42) were not significant predictors of internet-based economic returns. Model 1 explained 37% of the variation in dependent variables.

Model 2 added the influence of doctors’ service quality and mutual help groups on the revenue of doctors’ internet-based consultation based on model 1. According to the results shown in Table 3, *H1* doctors’ service quality had a positive effect on internet-based economic returns, and thus *H1* was supported (*P*<.001). The *H2* mutual help group did not have a positive effect on internet-based consultation income (*P*=.27). The degree of interaction and the level of 2-way communication between the service provider and the patient are critical to the customer’s ultimate perception of the service provider’s service results [38]. Thus, doctors’ replies are vital to patients as a result of these answers, which can alleviate distress. Model 2 explained 50.8% of the variation in the dependent variable. Compared with model 1, adding 2 tools of effort improved the explanatory ability of the model.

**Figure 2:**
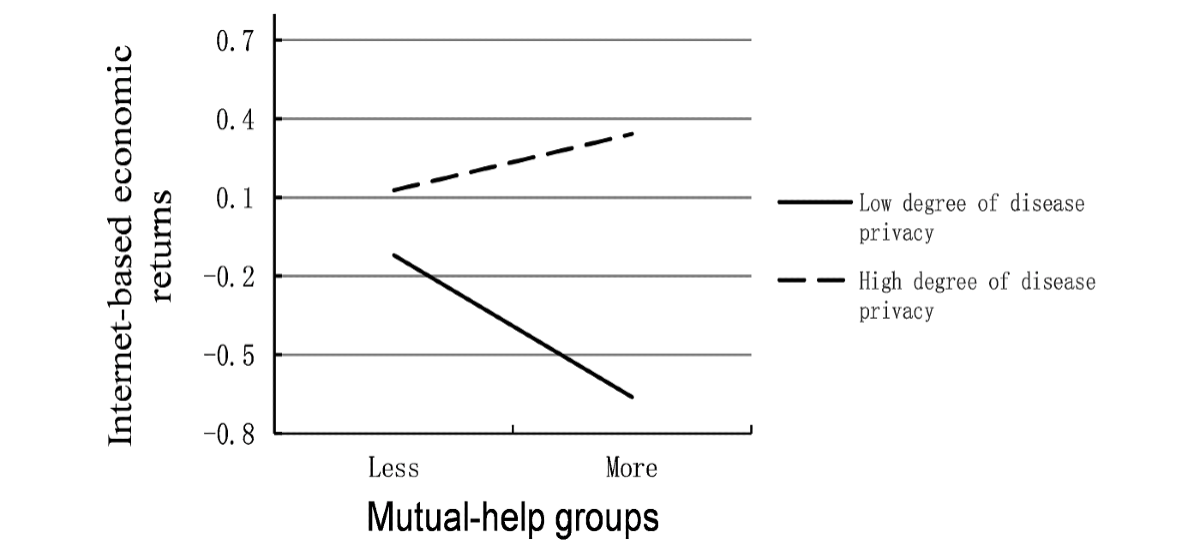
Interaction between mutual-help group and disease privacy.

Finally, we analyzed the effects of the 2 interaction terms, which are the interaction terms between doctors’ service quality and disease privacy, the interaction terms between mutual help group and disease privacy. The results indicate that *H3* is not supported (*P*=.16); however, *H4* is supported (*P*<.001). Privacy diseases can lead to social stigmatization, and these stigmatizations can have many negative effects on patients [39]. Although patients with privacy diseases are not willing to visit hospitals, they must receive doctors’ care, the concern of other people, and health care information to recover. Model 3 explained 58% of the variation in the dependent variable, which indicates that compared with model 2, the 2 interaction terms increased the explanatory power of the model. The interaction between mutual help groups and disease privacy is shown in Figure 2. When the degree of disease privacy increases, the influence of mutual help group on earnings changes from a negative correlation to a positive correlation, indicating that when patients consult low disease privacy, the more mutual help group, the lower the incomes; however, when patients consult high disease privacy, the more mutual help group, the higher the incomes.

## Discussion

### Principal Findings

The COVID-19 pandemic has significantly disrupted normal medical activities worldwide. To help decrease the spread of COVID-19, online health care platforms have been rapidly used by governments and users [40,41]. Currently, the Haodf website primarily provides 2 internet-based effort tools for doctors to interact with patients (doctors’ quality of service and mutual help groups). In this study, we sought to examine the effect of these tools on the economic returns of doctors’ internet-based consultations, and these mechanisms are adjusted by the degree of disease privacy. The primary findings of this study are summarized below.

#### Effect of Doctors’ Internet-Based Service Quality on Their Economic Return

Doctors’ internet-based service quality has a positive effect on the revenue of doctors’ internet-based consultation, which indicates that the high doctors service quality to patients’ consultation, the more economic return they obtain. In traditional Chinese culture, patients often judge the professional ability and service quality of doctors based on the hospital’s grade and the doctor’s status. Status is a sociological concept that focuses on rights or discrimination due to differences in social class rather than performance [42]. Patients have also become accustomed to long waiting times for outpatient registration, as Beijing has converged on the best resources in China. This situation causes many problems, including poor hospital environments and short communication between doctors and patients. Owing to COVID-19–related stay-at-home restrictions, in-person care is becoming less frequent. However, patients who do not leave home can use internet-based platforms for primary care in China. Thus, patients can save waiting time and enable better communication with high-status doctors from high-ranking hospitals. In addition, low-status doctors can put in their efforts in the OHC to earn more economic returns by patiently and carefully answering patients’ inquiries.

#### Effect of Mutual Help Groups on Doctors’ Economic Return

Mutual help groups did not affect their internet-based revenue; doctors could not obtain higher economic returns by building different types of topic groups. Whenever a given patient communicates with a doctor more than 3 times, the patient is automatically placed at the doctor’s request into different virtual communities. Owing to COVID-19–related stay-at-home restrictions, in-person communication among patients is less frequent [43,44]. Doctors put patients with the same disease into a virtual group that provides convenient communication among its members. However, patients are not willing to participate in group discussions too much, as the members in the virtual community feel strange and lack trust in each other. Thus, mutual help groups do not affect the revenue of internet-based consultations.

#### The Moderating Effect of Disease Privacy

Disease privacy positively regulates the influence of mutual help groups on the revenue of internet-based consultation, which indicates that when the degree of disease privacy increases, mutual help groups increase the benefits of doctors’ internet-based consultation. Patients with nonprivacy diseases easily communicate in person with others without any concerns. These patients can also communicate offline with patients who have similar diseases without the fear of discrimination or unfair treatment [45]; thus, they do not have strong intentions to find companions to communicate about their diseases in a virtual group. Patients with nonprivacy diseases in a virtual community were unfamiliar with each other, had a strong sense of strangeness, and could not trust each other. When patients have not joined a virtual community on their own [6], doctors’ efforts would not be recognized by patients; therefore, the economic return would not increase. Conversely, patients with privacy diseases are reluctant to go to hospitals for consultation because of the risk to their privacy; thus, they have few opportunities to meet people who have a similar disease. In this situation, patients may not receive efficient or timely medical treatment because of a lack of medical knowledge and communication with doctors. In virtual communities, doctors build a bridge between patients with privacy diseases and others in the outside world, and members in the mutual help group do not know each other; thus, users do not need to worry about disclosure of their disease status. Patients with privacy diseases were more willing to communicate with members of the group. Thus, doctors’ efforts to build mutual help groups can be recognized by patients, which leads to improved economic returns.

### Limitations

This study has produced some beneficial results; however, there are still certain limitations that must be addressed in future studies. First, data were taken from one OHC, the Good Doctor website, which is the most acceptable web-based consultation platform [33,46]; thus, the universality of the results is questionable. Future studies will consider collecting physician data from several different types of OHCs and empirically testing the model’s findings. Second, in this study, only two tools (doctors’ service quality and mutual help groups) provided by a single health consultation platform to describe the interaction efforts between doctors and patients were included, which may lead to an insignificant influence of the tools on the doctors’ economic returns. In the future, the interactive efforts of other health consultation websites should be considered. Third, only one type of privacy disease was investigated (hepatitis B). Patients with different diseases have different degrees of involvement in information processing, and the degree of influence of information on patients’ choices is also different [47-49]. Although the representativeness of this disease was considered in the selection of this study, further studies should consider diseases with different privacy levels, as different diseases that need privacy may have different service requirements.

### Conclusions

In the face of the COVID-19 pandemic, internet-based medical treatment has become a new choice for people seeking medical treatment because of its unique features, including a lack of geographical restrictions, no in-person contact, and prevention of infection. Governments have rapidly used alternative methods for health care delivery, including web-based consultation, when people are restricted from their normal activities [5,8]. Many internet consultation platforms, including Haodf website, have seen a surge in visits during the epidemic. The prosperity of the platform depends on the continuous participation and efforts of doctors, and obtaining satisfactory economic returns is an important motivation for doctors’ continuous participation. Therefore, it is of great significance for platforms, doctors, and patients to study internet-based consultation income.

This study expands and enriches the application and connotations of individual efforts. Individual effort has been focused on traditional industries and has never been used in online health care services. Online health services differ from traditional offline industries. This study expanded the application scope of efforts from the traditional offline industry to online health services and further identified 2 types of effort tools (doctors’ replies and mutual help groups) based on the characteristics of the internet-based health market. These 2 effort tools are unique variables in the field of eHealth and have not been covered in previous studies in the field of electronic commerce. In addition, the degree of disease privacy is a unique variable in the field of OHCs, which has not been mentioned in previous studies in the field of electronic services, including electronic government and electronic commerce. This study identified that the uncertainty of privacy disclosure and the uncertainty of service quality may increase when the degree of disease privacy improves; thus, doctors must put in more efforts online to improve their services. Concurrently, this study also clearly distinguished the different influence mechanisms of the degree of disease privacy on the doctors’ service quality and the mutual help groups. Doctors who built many mutual help groups could obtain higher incomes when serving patients with privacy diseases.

## Abbreviations

OHC: online health care community
VIF: variance inflation factor
